# The diagnostic value of combined *Aspergillus* IgG antibody and galactomannan antigen detection in nonneutropenic patients with pulmonary aspergillosis

**DOI:** 10.3389/fmed.2025.1652051

**Published:** 2025-09-08

**Authors:** Junli Zhang, Wei Ren, Hong Deng, Ying Zhang, Meili Gong, Liyan Bo, Baining Li, Congcong Li

**Affiliations:** ^1^Department of Respiratory and Critical Care Medicine, General Hospital of Northern Theater Command, Shenyang, China; ^2^Department of Clinical Laboratory, General Hospital of Northern Theater Command, Shenyang, China; ^3^The 968th Hospital of the Joint Logistics Support Force of the Chinese People’s Liberation Army, Jinzhou, China

**Keywords:** pulmonary *Aspergillus* infection, *Aspergillus* antibody IgG, galactomannan antigen assay, diagnosis, nonneutropenic

## Abstract

**Introduction:**

To evaluate the diagnostic efficacy of combined *Aspergillus*-specific IgG antibody and galactomannan antigen testing in nonneutropenic patients with pulmonary aspergillosis.

**Methods:**

A total of 561 patients were included in the dataset for the analysis.

**Results:**

The Asp IgG positivity rate was significantly greater in participants ultimately diagnosed with pulmonary aspergillosis than in the control group (*p* < 0.01). *Aspergillus* antibody IgG had diagnostic significance for pulmonary aspergillosis (*p* < 0.01), with an AUC of 0.748 (or 0.738 excluding ABPA), *p* < 0.01. The optimal diagnostic cut-off for *Aspergillus* antibody IgG was ≥102.95 U/L. The results of the combined analysis of *Aspergillus* antibody IgG and the GM test revealed that negative test results for both *Aspergillus* antibody IgG and GM could significantly increase the diagnostic specificity, which could reach 98.3% (or 97.2% excluding ABPA).

**Discussion:**

In conclusion, for clinical patients without neutropenia, *Aspergillus* antibody IgG in the peripheral blood is a valuable diagnostic method for pulmonary *Aspergillus* infection. Combining this method with the GM test can significantly increase diagnostic specificity.

## 1 Introduction

Pulmonary aspergillosis (PA) traditionally affects patients with profound neutropenia, prolonged immunosuppressive therapy, or solid organ transplantation. Unlike bacterial infections, PA remains inherently challenging to diagnose due to the difficulty of obtaining direct evidence of fungal invasion, such as histological demonstration of hyphae or positive *Aspergillus* cultures (1). Current diagnostic algorithms rely heavily on serological biomarkers, including galactomannan (GM) antigen, (1–3)-β-D-glucan (G test), and molecular methods such as *Aspergillus* DNA polymerase chain reaction (PCR) (1, 2). For high-risk groups, clinicians often initiate pre-emptive or empirical antifungal therapy based on clinical features, radiographic findings, and serological trends (2).

PA is a lethal fungal infection. Traditionally, most PA cases are thought to occur in neutropenic patients. Recent epidemiological shifts have drawn increasing attention to PA in nonneutropenic patients. Recent studies have shown that the proportion of nonneutropenic patients is gradually increasing. Globally, approximately 7 to 24% of patients with bronchiectasis will develop *Aspergillus* colonization or infection. During the influenza and COVID-19 pandemics, the incidence of nonneutropenic PA rose significantly, accounting for 10–20% of all invasive fungal infections (2) and contributing to increased mortality (3). Unlike immunocompromised populations, nonneutropenic patients require rigorous diagnostic criteria before antifungal initiation due to the potential risks of overtreatment. Avoiding delays in diagnosis and treatment are particularly critical, as studies on influenza-associated PA (IAPA) have reported mortality rates of 56% with timely intervention versus 71% when treatment is delayed (2, 4).

Distinguishing between colonization, infection, and contamination is the key to timely diagnosis, which is highly important for providing early treatment and reducing the mortality rate. However, in non-neutropenic patients, the diagnose of pulmonary aspergillosis is a challenge. First, the classical radiological features of PA showed are often absent in these patients. Second, obtaining samples for histology by biopsy of infected tissue is often not feasible or delayed. Owing to the great difficulty of pathological and microbiological diagnosis, serological antibody and antigen examinations have naturally become important indicators for pre-emptive treatment. Although current guidelines have corresponding recommendations for the diagnostic efficacy of Aspergillus antibody IgG and the GM antigen test in pulmonary Aspergillus infection (5), their application in the diagnosis of pulmonary Aspergillus infection in nonneutropenic patients still requires in-depth research to explore the value of the combined application of *Aspergillus* antibody IgG and the GM antigen test.

## 2 Research subjects and methods

### 2.1 Research subjects

This retrospective study included patients who were hospitalized in the Department of Respiratory and Critical Care Medicine of the General Hospital of the Northern Theater Command and who underwent *Aspergillus* antibody IgG and GM tests from January 1, 2020, to December 31, 2024. There are 125 beds in this center, and 17,111 patients hospitalized from January 1, 2020 to December 31, 2024 in total. This study was approved by the hospital ethics committee, and the ethics approval number is Y (2025) No. 102. The inclusion criteria were a. patients had risk factors for PA or ABPA, experienced a failure of empiric antimicrobial therapy, or patients’ chest CT scan showed signs of *Aspergillus* infection, such as pulmonary nodules, consolidation, infiltrates or cavity; b. received GM or *Aspergillus* IgG test. And the exclusion criteria were a. peripheral blood neutrophil count was <0.5*109/L; b. asymptomatic *Aspergillus* colonization.

**Figure 1:**
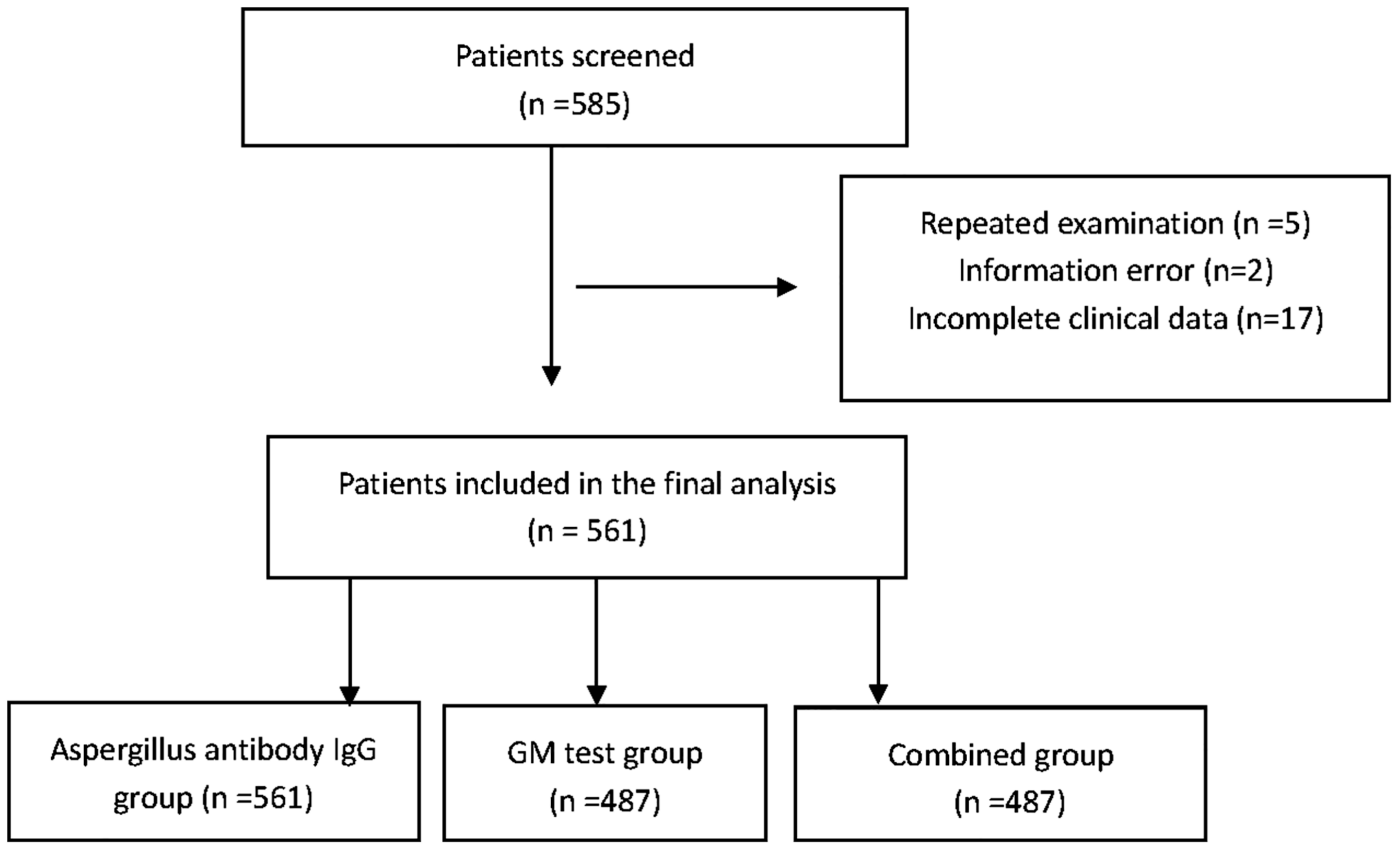
Flow chart of research subjects enrolment and grouping.

In total, we screened 585 patients. After the exclusion of 5 patients with repeated examinations in a short period, 2 patients with information errors, and 17 patients with incomplete clinical data, 561 patients were ultimately included in the analysis. The patients were divided into three subgroups for analysis (see Figure 1). Among them, 561 patients were in the *Aspergillus* antibody IgG group, 487 patients were in the GM test group, and 487 patients were in the group receiving combined *Aspergillus* antibody IgG and GM tests.

### 2.2 Procedures

*Aspergillus* antibody IgG was detected via an *Aspergillus* antibody IgG detection kit produced by Danna (Tianjin) Biotechnology Co., Ltd. The positive cut-off point was ≥100 U/L. The GM test was carried out with an *Aspergillus* galactomannan detection kit from the same manufacturer. The positive cut-off point was ≥0.5 μg/L for peripheral blood and ≥1 μg/L for bronchoalveolar lavage fluid. For the diagnosis of pulmonary aspergillosis, we adopted the modified AspICU algorithm (6). Based on symptoms, signs, imaging findings, laboratory tests (especially microbiological and/or pathological examination results), and treatment outcomes, two experts in respiratory medicine independently diagnosed the patients. In cases where the experts had conflicting opinions, a consensus diagnosis was reached through discussion. By comparing the results of the *Aspergillus* antibody IgG test and the GM test, it was determined whether they were consistent with the diagnosis. Thus, we obtained the sensitivity and specificity of the *Aspergillus* antibody IgG test and the GM test in diagnosing pulmonary *Aspergillus* infection, as well as the clinical diagnostic value of using the two indicators in combination.

### 2.3 Statistical analysis

Measurement data are expressed as the means ± standard deviations (x̄ ± s). The *t* test was used for the analysis of differences between groups of continuous measurement data, and the chi-square test (*χ*^2^ test) was used for the analysis of differences between groups of count data. If the analysis result was *p* < 0.05, the difference was considered statistically significant. For the analysis of sensitivity and specificity, SPSS 23 statistical analysis software was used for regression analysis, calculation of the area under the ROC curve, and determination of the optimal cut-off point.

## 3 Results

### 3.1 Demographic data

Among the 561 patients, there were 330 males and 231 females, with an average age of 61.6 ± 14.06 years. There were 561 patients in the *Aspergillus* antibody IgG group, including 330 males and 231 females, with an average age of 61.6 ± 14.06 years. In both the GM test group and the combined group, there were 487 patients, including 291 males and 196 females, with an average age of 61.5 ± 14.2 years (see Table 1).

**Table 1:** Demographic data.

Group	Male	Female	Age (years)
*Aspergillus* antibody IgG group	330	231	61.6 ± 14.06
GM test group	291	196	61.5 ± 14.2
Combined group	291	196	61.5 ± 14.2

### 3.2 Value of *Aspergillus* antibody IgG in the diagnosis of pulmonary *Aspergillus* infection

Among the 561 patients included in the analysis, 60 were diagnosed with pulmonary *Aspergillus* infection, 11 were diagnosed with ABPA, and 490 were diagnosed with neither pulmonary *Aspergillus* infection nor ABPA. Among the 60 patients with pulmonary *Aspergillus* infection, 40 had positive peripheral blood *Aspergillus* antibody IgG, and 20 had negative results; among the 11 ABPA patients, 8 had positive peripheral blood *Aspergillus* antibody IgG, and 3 had negative results; among the 490 patients with neither pulmonary *Aspergillus* infection nor ABPA, 87 had positive peripheral blood *Aspergillus* antibody IgG, and 403 had negative results (see Table 2). If ABPA patients were included in pulmonary *Aspergillus* infection, the positivity rate for peripheral blood *Aspergillus* antibody IgG was significantly greater than that in patients without pulmonary *Aspergillus* infection (*χ*^2^ = 98.92, *p* < 0.01), with a sensitivity of 67.6% and a specificity of 82.2%. If ABPA patients were included in those without pulmonary *Aspergillus* infection, the positivity rate for peripheral blood *Aspergillus* antibody IgG was significantly greater than that in patients without pulmonary *Aspergillus* infection (*χ*^2^ = 66.73, *p* < 0.01), with a sensitivity of 66.7% and a specificity of 81.0%.

**Table 2:** Value of *Aspergillus* antibody IgG in the diagnosis of pulmonary *Aspergillus* infection.

Items	Pulmonary *Aspergillus* infection (including ABPA)	Non-pulmonary *Aspergillus* infection (including ABPA)	Pulmonary *Aspergillus* infection (excluding ABPA)	Non-pulmonary *Aspergillus* infection (excluding ABPA)
Positive cases (*n*)	48	87	40	95
Negative cases (*n*)	23	403	20	406
Chi-square value	98.92		66.73	
*p*-value	*p* < 0.01		*p* < 0.01	
Sensitivity (%)	67.6		66.7	
Specificity (%)	82.2		81.0	

### 3.3 Value of the GM test in the diagnosis of pulmonary *Aspergillus* infection

Among the 487 patients included in the analysis, 54 were diagnosed with pulmonary *Aspergillus* infection, 11 were diagnosed with ABPA, and 422 were diagnosed with neither pulmonary *Aspergillus* infection nor ABPA. Among the 54 patients with pulmonary *Aspergillus* infection, 23 had positive GM test results in peripheral blood and/or bronchoalveolar lavage fluid, and 31 had negative results; among the 11 ABPA patients, 5 had positive GM test results in peripheral blood and/or bronchoalveolar lavage fluid, and 6 had negative results; among the 422 patients with neither pulmonary *Aspergillus* infection nor ABPA, 73 had positive GM test results in peripheral blood and/or bronchoalveolar lavage fluid, and 349 had negative results (see Table 3). If ABPA patients were included in pulmonary *Aspergillus* infection, the GM positivity rate in peripheral blood and/or bronchoalveolar lavage fluid was significantly greater than that in patients without pulmonary *Aspergillus* infection (*χ*^2^ = 22.77, *p* < 0.01), with a sensitivity of 43.1% and a specificity of 82.7%. If ABPA patients were included in those without pulmonary *Aspergillus* infection, the GM positivity rate in peripheral blood and/or bronchoalveolar lavage fluid was significantly greater than that in patients without pulmonary *Aspergillus* infection (*χ*^2^ = 17.64, *p* < 0.01), with a sensitivity of 42.6% and a specificity of 82.0%.

**Table 3:** Value of the GM Test in the diagnosis of pulmonary *Aspergillus* infection.

Items	Pulmonary *Aspergillus* infection (including ABPA)	Non-pulmonary *Aspergillus* infection (including ABPA)	Pulmonary *Aspergillus* infection (excluding ABPA)	Non-pulmonary *Aspergillus* infection (excluding ABPA)
Positive cases (*n*)	28	73	23	78
Negative cases (*n*)	37	349	31	355
Chi-square value	22.77		17.64	
*p*-value	*p* < 0.01		*p* < 0.01	
Sensitivity (%)	43.1		42.6	
Specificity (%)	82.7		82.0	

### 3.4 Value of copositive results of *Aspergillus* antibody IgG and GM tests in the diagnosis of pulmonary *Aspergillus* infection

Among the 487 patients included in the analysis, 54 were diagnosed with pulmonary *Aspergillus* infection, 11 were diagnosed with ABPA, and 422 were diagnosed with neither pulmonary *Aspergillus* infection nor ABPA. Among the 54 patients with pulmonary *Aspergillus* infection, 14 had positive results for both peripheral blood *Aspergillus* antibody IgG and GM, and 40 had negative results; among the 11 ABPA patients, 5 had positive results for both peripheral blood *Aspergillus* antibody IgG and GM, and 6 had negative results; among the 422 patients with neither pulmonary *Aspergillus* infection nor ABPA, 7 had positive results for both peripheral blood *Aspergillus* antibody IgG and GM, and 415 had negative results (see Table 4). If ABPA patients were included in pulmonary *Aspergillus* infection, the correlation rate between peripheral blood *Aspergillus* antibody IgG and GM positivity was significantly greater than that in patients without pulmonary *Aspergillus* infection (*χ*^2^ = 84.73, *p* < 0.01), with a sensitivity of 29.2% and a specificity of 98.3%. If ABPA patients were included in those without pulmonary *Aspergillus* infection, the correlation rate of positive peripheral blood *Aspergillus* antibody IgG and GM tests was significantly greater than that in patients without pulmonary *Aspergillus* infection (*χ*^2^ = 50.93, *p* < 0.01), with a sensitivity of 25.9% and a specificity of 97.2%.

**Table 4:** Value of the combined *Aspergillus* antibody IgG and GM tests in the diagnosis of pulmonary *Aspergillus* infection.

Items	Pulmonary *Aspergillus* infection (including ABPA)	Non-pulmonary *Aspergillus* infection (including ABPA)	Pulmonary *Aspergillus* infection (excluding ABPA)	Non-pulmonary *Aspergillus* infection (excluding ABPA)
Positive cases (*n*)	19	7	14	12
Negative cases (*n*)	46	415	40	421
Chi-square value	84.73		50.93	
*p*-value	*p* < 0.01		*p* < 0.01	
Sensitivity (%)	29.2		25.9	
Specificity (%)	98.3		97.2	

### 3.5 Value of positive results of *Aspergillus* antibody IgG or GM tests in the diagnosis of pulmonary *Aspergillus* infection

Among the 487 patients included in the analysis, 54 were diagnosed with pulmonary *Aspergillus* infection, 11 were diagnosed with ABPA, and 422 were diagnosed with neither pulmonary *Aspergillus* infection nor ABPA. Among the 54 patients with pulmonary *Aspergillus* infection, 40 had positive test results for either *Aspergillus* antibody IgG or GM, and 14 had negative results; among the 11 ABPA patients, 8 had positive test results for either *Aspergillus* antibody IgG or GM, and 3 had negative results; among the 422 patients with neither pulmonary *Aspergillus* infection nor ABPA, 135 had positive test results for either *Aspergillus* antibody IgG or GM, and 287 had negative results (see Table 5). If ABPA patients were included in pulmonary *Aspergillus* infection, the rate of positivity for either peripheral blood *Aspergillus* antibody IgG or GM was significantly greater than that in patients without pulmonary *Aspergillus* infection (*χ*^2^ = 213.77, *p* < 0.01), with a sensitivity of 73.8% and a specificity of 68.0%. If ABPA patients were included in those without pulmonary *Aspergillus* infection, the rate of positivity for either peripheral blood *Aspergillus* antibody IgG or GM was significantly greater than that in patients without pulmonary *Aspergillus* infection (*χ*^2^ = 34.49, *p* < 0.01), with a sensitivity of 74.1% and a specificity of 67.0%.

**Table 5:** Value of the combined *Aspergillus* antibody IgG and GM tests in the diagnosis of pulmonary *Aspergillus* infection.

Items	Pulmonary *Aspergillus* infection (including ABPA)	Non-pulmonary *Aspergillus* infection (including ABPA)	Pulmonary *Aspergillus* infection (excluding ABPA)	Non-pulmonary *Aspergillus* infection (excluding ABPA)
Positive cases (*n*)	48	135	40	143
Negative cases (*n*)	17	287	14	290
Chi-square value	213.77		34.49	
*p*-value	*p* < 0.01		*p* < 0.01	
Sensitivity (%)	73.8		74.1	
Specificity (%)	68.0		67.0	

### 3.6 Regression analysis

#### 3.6.1 Diagnostic value of *Aspergillus* antibody IgG (ABPA included in pulmonary *Aspergillus* infection)

**Figure 2:**
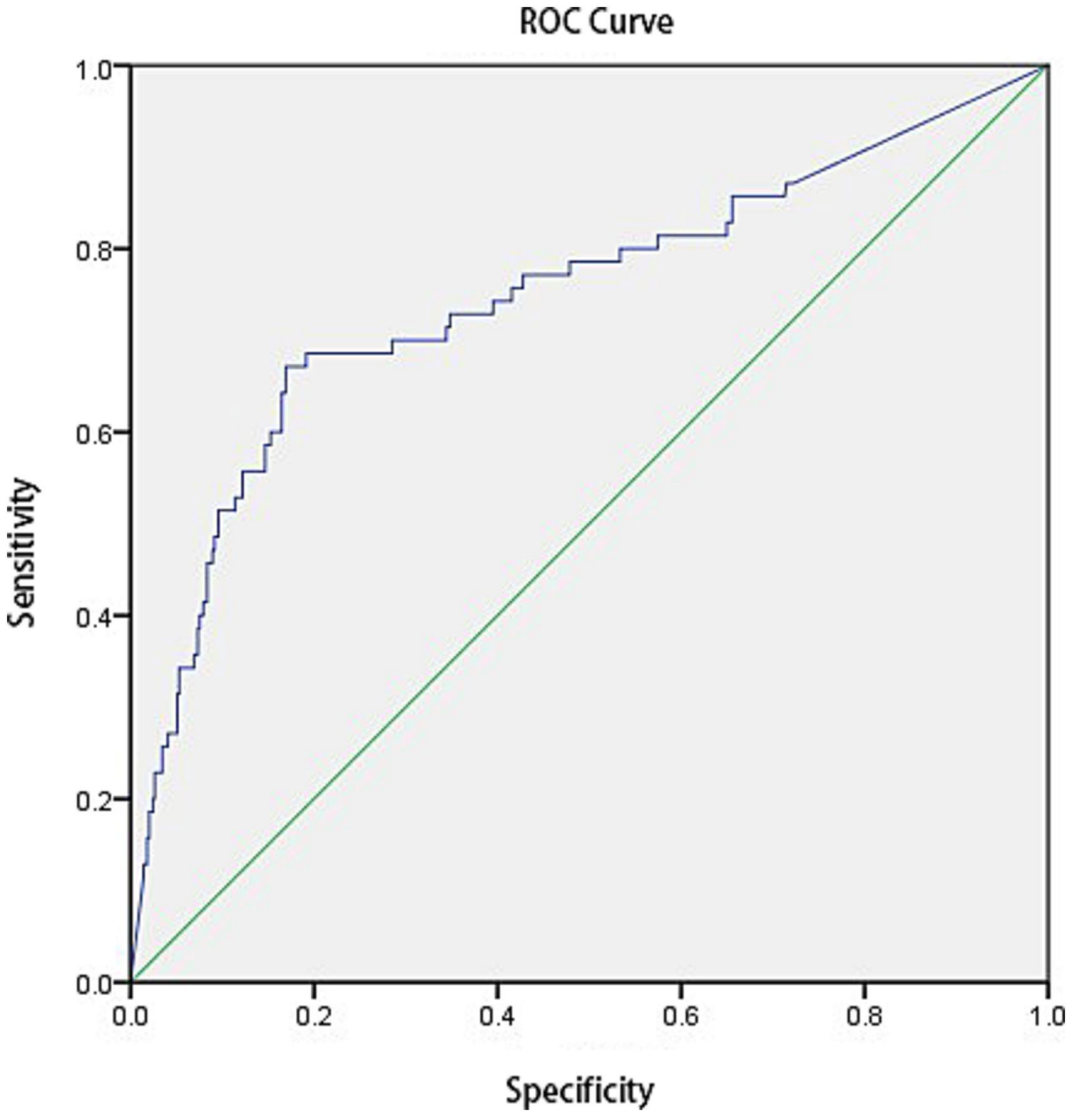
Diagnostic value of *Aspergillus* antibody IgG (ABPA included in pulmonary *Aspergillus* infection).

Binary logistic analysis revealed that *Aspergillus* antibody IgG had diagnostic significance for pulmonary *Aspergillus* infection (*p* < 0.01), with an AUC of 0.748 (*p* < 0.01) (see Figure 2). According to the results of the Youden index analysis, the optimal diagnostic cut-off for *Aspergillus* antibody IgG was ≥102.95 U/L.

#### 3.6.2 Diagnostic value of *Aspergillus* antibody IgG (ABPA not included in pulmonary *Aspergillus* infection)

**Figure 3:**
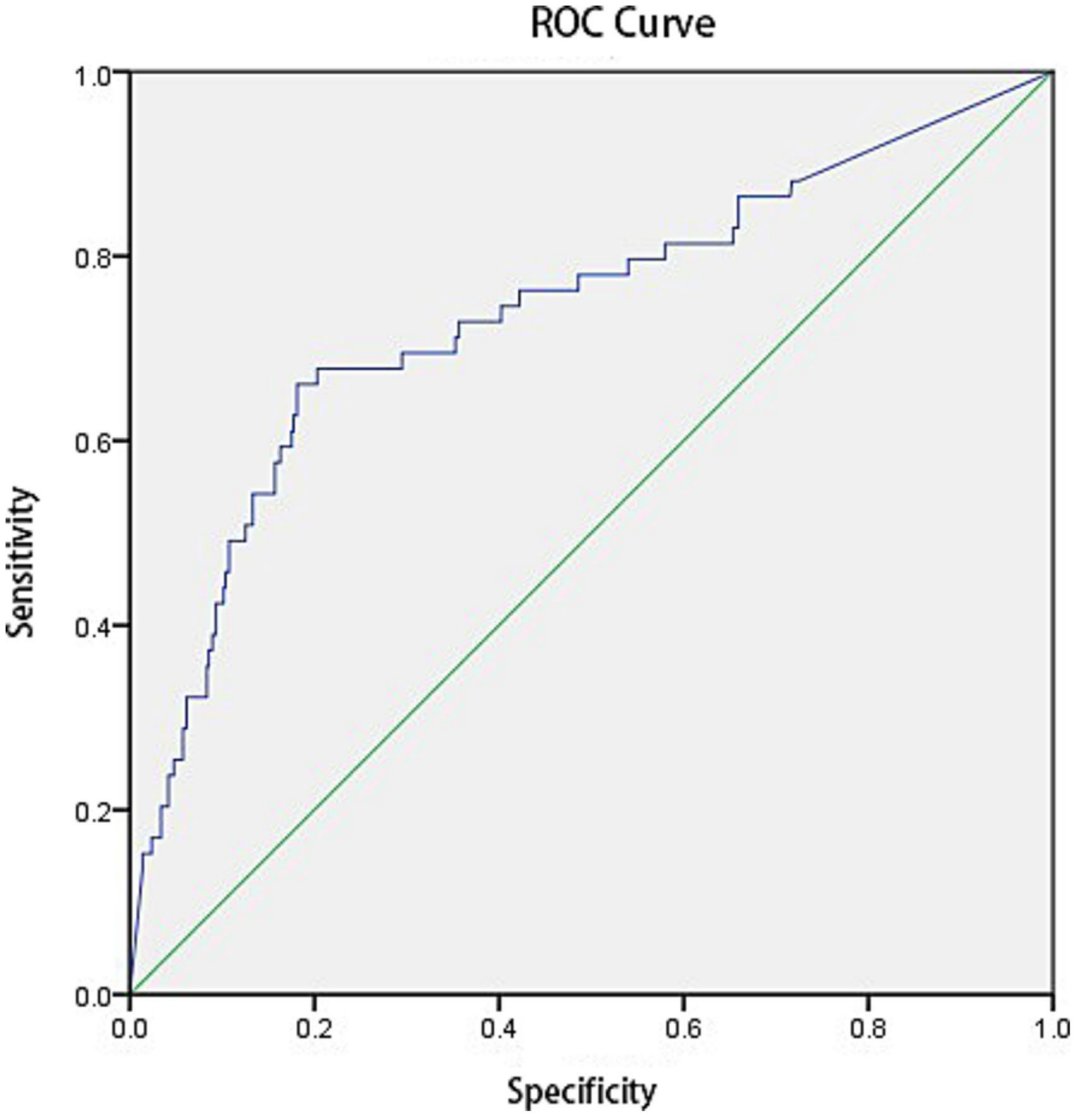
Diagnostic value of *Aspergillus* antibody IgG (ABPA not included in pulmonary *Aspergillus* infection).

Binary logistic analysis revealed that *Aspergillus* antibody IgG had diagnostic significance for pulmonary *Aspergillus* infection (*p* < 0.01), with an AUC of 0.738 (*p* < 0.01) (see Figure 3). According to the results of the Youden index analysis, the optimal diagnostic cut-off for *Aspergillus* antibody IgG was ≥102.95 U/L.

## 4 Discussion

Invasive pulmonary aspergillosis (IPA) is a lethal fungal infection with a mortality rate of approximately 30–80% (7, 8). In patients with ARDS, the presence of pulmonary *Aspergillus* infection can lead to excess mortality rates ranging from 16 to 25% compared with patients without evidence of aspergillosis (9). The epidemiological characteristics of IPA are undergoing important changes. Traditionally, most IPA cases are thought to occur in neutropenic patients (10). However, recent studies have shown that the proportion of nonneutropenic patients is gradually increasing (8, 10). This change is closely related to structural lung diseases such as chronic obstructive pulmonary disease (COPD), asthma, rheumatoid polyarthritis, giant cell arteritis, and vasculitis (11, 12). Globally, approximately 7–24% of patients with bronchiectasis will develop *Aspergillus* colonization or infection (13, 14). Distinguishing between colonization, infection, and contamination is the key to timely diagnosis, which is highly important for providing early treatment and reducing the mortality rate. In terms of diagnostic strategy, although histopathological examination is the gold standard for diagnosing pulmonary *Aspergillus* infection, its clinical application is limited by the risks of invasive procedures such as the complication rate of thoracoscopy/percutaneous lung biopsy is approximately 5–15%, and it is difficult to obtain ideal specimens. Microbiological smears and cultures are also highly important for diagnosis. In terms of microbiological testing, the sensitivity of bronchoalveolar lavage fluid (BALF) culture is less than 50% (15), and it is very likely to fail because of contamination, affecting the efficiency and accuracy of rapid diagnosis.

These problems have made serological testing an important supplement in the diagnosis of invasive pulmonary *Aspergillus* infection. The guidelines of the European Confederation of Medical Mycology (ECMM) clearly recommend that *Aspergillus* IgG antibodies be used as one of the preferred screening indicators for chronic pulmonary aspergillosis (CPA) (16, 17). However, regarding its value in acute/subacute IPA, its diagnostic significance still needs to be explored. Existing studies show that there are differences in sensitivity, which may be related to the setting of the detection threshold. In nonneutropenic patients, pulmonary *Aspergillus* infection can significantly increase the positive rate of *Aspergillus* antibody IgG in the peripheral blood by activating the human immune response (18–20). Moreover, the cut-off point for diagnosing pulmonary *Aspergillus* infection with *Aspergillus* antibody IgG in the peripheral blood is still being discussed and researched (21, 22).

ABPA is a special type of pulmonary *Aspergillus* infection. Theoretically, it is not invasive pulmonary aspergillosis but is also a disease caused by *Aspergillus* infection of the bronchi, leading to allergic reactions. Some research results have shown that *Aspergillus* antibody IgG has a certain value in the diagnosis of this disease (23–25). The GM test of peripheral blood and bronchoalveolar lavage fluid is also a common method for detecting *Aspergillus* antigen, but its actual diagnostic value in clinical practice is still controversial (26, 27).

The results of this study show that *Aspergillus* antibody IgG in peripheral blood has certain value in the diagnosis of nonneutropenic pulmonary *Aspergillus* infection. If ABPA patients are included in pulmonary *Aspergillus* infection, the positivity rate for *Aspergillus* antibody IgG in the peripheral blood was significantly greater than that in patients without pulmonary aspergillosis infection (*p* < 0.01), with a sensitivity of 67.6% and a specificity of 82.2%. If ABPA patients are included in those without pulmonary aspergillosis infection, the positivity rate for *Aspergillus* antibody IgG in the peripheral blood was significantly greater than that in patients without pulmonary aspergillosis infection (*p* < 0.01), with a sensitivity of 66.7% and a specificity of 81.0%.

This study revealed through binary logistic regression analysis that when the IgG threshold is set at 102.95 U/L, regardless of whether ABPA is included, its diagnostic efficacy (AUC 0.738–0.748) is basically consistent with the results of many previous studies (AUC 0.71) (28, 29). The optimized Asp IgG cut-off (≥102.95 U/L) addresses critical gaps in PA diagnostics for nonneutropenic patients by standardizing result interpretation, balancing sensitivity and specificity, and enabling effective combined testing.

Notably, the IgG level in ABPA patients often exceeds 150 IU/L (20, 30), which explains the impact of the adjustment of the ABPA group in this study on the specificity (from 82.2 to 81.0%). In terms of antigen detection, the GM test results are consistent with those of a meta-analysis (combined sensitivity of 53% and specificity of 94%) (31). However, it should be noted that, according to the results of this study (sensitivity of 43.1%), the sensitivity of BALF-GM detection may be reduced in nonneutropenic patients.

The optimization of the combined detection strategy is an important finding of this study. When the strategy of “diagnosis when either IgG or GM is positive” is adopted, the sensitivity is increased to 73.8% (95% CI 65.2–81.1), and the strategy of “excluding diagnosis when both are negative” brings the specificity to 98.3%, which is comparable to that of the method of combining the GM test with PCR (specificity of 98%) (32). This result, which is close to 100%, has significant clinical value. It can help us exclude the possibility of *Aspergillus* infection in some patients. This finding supports the recommendation for the application of the multiparameter diagnostic model in the joint ECMM/ERS statement and provides an important evidentiary basis for the application of the ECMM/ERS guidelines in atypical populations such as nonneutropenic patients (5).

In terms of the limitations of this study, the inherent bias of the retrospective design, such as incomplete records of antibiotic use, may affect the reliability of the results. In the future, multicentre prospective studies should be conducted and new molecular diagnostic technologies, such as quantitative PCR, should be included for comparative analysis.

## 5 Conclusion

In clinical patients without neutropenia, *Aspergillus* antibody IgG in the peripheral blood is a valuable diagnostic method for pulmonary *Aspergillus* infection. The combination of *Aspergillus* antibody IgG and GM tests can significantly increase diagnostic specificity. Negative results on both the *Aspergillus* antibody IgG and GM tests have great value in excluding pulmonary *Aspergillus* infection.

## Data Availability

The raw data supporting the conclusions of this article will be made available by the authors, without undue reservation.
